# Supplementary material for: Prevalence and its associated factors of medical error reporting among healthcare professionals in Ethiopia: Systematic review and meta-analysis
Source: PLoS One. 2025 Jun 2;20(6):e0325114. doi: 10.1371/journal.pone.0325114 (PMC12129177; doi:10.1371/journal.pone.0325114)
Supplement: S2 File — (DOCX) [file pone.0325114.s002.docx]

**Supplemental file 2**: Search strategy

The adopted CoCoPoP format was used to retrieve relevant studies. The CoCoPoP consists of

Condition (Co), context (Co), and population (Pop) as described below.

**A. Condition**: prevalence of medical error reporting.

**B. Context**: Ethiopia.

**C. Populations**: Healthcare professionals, healthcare workers.

Using the above CoCoPoP, we constructed the following review questions which focused on retrieving relevant studies.

1. What is the prevalence medical error reporting among healthcare professionals in Ethiopia?

2. What are the factors affecting medical error reporting among healthcare professionals in Ethiopia?

The primary studies were subsequently retrieved from the Google Scholar, PubMed, and Web of Science databases using the following keyword and MeSH terms: "Medical errors" AND "Reporting" OR "disclosure" AND "magnitude" AND "associated factors" OR "predictors" OR "determinants" AND "healthcare professionals" OR "nurses" OR "doctors" OR "pharmacists" AND "Ethiopia". The search string was developed using ʺANDʺ and ʺORʺ Boolean operators.

| Databases | Key search terms or phrases |
| --- | --- |
| Google Scholar | "Medical errors" AND "Reporting" OR "disclosure" AND "magnitude" AND "associated factors" OR "predictors" OR "determinants" AND "healthcare professionals" OR "nurses" OR "doctors" OR "pharmacists" AND "Ethiopia" |
| Total articles | 500 |
| Manual Search and university repository | Prevalence of medical error reporting and associated factors among health professionals in Ethiopia |
| Total articles | 23 |
| PubMed | "Medical errors" AND "Reporting" OR "disclosure" AND "magnitude" AND "associated factors" OR "predictors" AND "healthcare professionals" AND "Ethiopia" |
| Total articles | 510 |
| Web of Science | "Medical errors" AND "Reporting" OR "disclosure" AND "magnitude" AND "associated factors" OR "predictors" OR "determinants" AND "healthcare professionals" AND "Ethiopia" |
| Total Articles | 200 |
| Total retrieved articles from all databases | 1,233 |
