# Supplementary material for: Prevalence and its associated factors of medical error reporting among healthcare professionals in Ethiopia: Systematic review and meta-analysis
Source: PLoS One. 2025 Jun 2;20(6):e0325114. doi: 10.1371/journal.pone.0325114 (PMC12129177; doi:10.1371/journal.pone.0325114)
Supplement: S3 File — (DOCX) [file pone.0325114.s003.docx]

**Supplementary file 3** a list of excluded and included articles on a study of medical error reporting and associated factors among healthcare professionals in Ethiopia. Systematic Review and Meta-analysis.

| First Author/publication year/ | Reason of exclusion and inclusion |
| --- | --- |
| 1.(Ebrahimipour, Mahmoudian et al. 2016) | Study area |
| 2.(Holmström, Laaksonen et al. 2015) | Title and study area |
| 3.(Yorke, Dang et al. 2016) | Title and area of study |
| 4.(Richter 2013) | Title and outcome |
| 5.(Petrie, Anton et al. 2016) | Title, study design and study setting |
| 6.(Uwimana 2017) | Study area |
| 7.(Naothavorn, Puranitee et al. 2023) | Study design and setting |
| 8.(Nsimenta 2022) | Title, outcome and study area |
| 9.(AHMAD) | Title and study area |
| 10.(Feleke, Mulatu et al. 2015) | Title and outcome |
| 11.(Wondmieneh, Alemu et al. 2020) | Title and abstract |
| 12.(Bante, Mersha et al. 2023) | Title and outcome |
| 13.(Tsegaye, Alem et al. 2020) | Title and outcome |
| 14.(Alemu, Belachew et al. 2017) | Title and abstract |
| 15.(Mekonen, Gebrie et al. 2020) | Title, outcome and abstract |
| 16.(Alebachew, Tsegaye et al. 2020) | Title and outcome |
| 17.(Firde 2023) | Title and abstract |
| 18.(Gebremariam, Sema et al. 2023) | Title and outcome |
| 19.(Assefa, Woldie et al. 2012) | Abstract and outcome |
| 20.(Mohammed, Mahmud et al. 2022) | Title and abstract |
| 21.(Feyissa, Kebede et al. 2020) | Title and study population |
| 22.(Dedefo, Mitike et al. 2016) | Title and outcome |
| 23. (Baraki, Abay et al. 2018) | Title and abstract |
| 24.(Gebre, Addisu et al. 2021) | Title, study design and population |
| 25.(Tassew, Feleke et al. 2022) | Title and abstract |
| 26.(Gebrye, Wudu et al. 2023) | Title and abstract |
| 27.(Mohammed, Taddele et al. 2021) | Title and outcome |
| 28.(Degfe and Mohammed 2023) | Title and abstract |
| 29.(Yayehrad, Getachew et al. 2024) | Title, outcome and study population |
| 30.(Dorothy, Yadesa et al. 2021) | Study area, title and outcome |
| 31.(Haile, Takele et al. 2020) | Title and abstract |
| 32.(Abebe, Alemayehu et al. 2024) | Title and outcome |
| 33.(Bifftu and Mekonnen 2020) | Title and abstract |
| 34.(Kebede and Kefale 2019) | Study population, title and outcome |
| 35.(Zeleke, Chanie et al. 2014) | Title and outcome |
| 36.(Sahilu, Getachew et al. 2020) | Study population and outcome |
| 37.(Assefa, Teferi et al. 2023) | Title and outcome |
| 38.(Adem, Abdela et al. 2021) | Title and abstract |
| 39.(Berhan, Malede et al. 2021) | Outcome, abstract and title |
| 40.(Wake, Tuji et al. 2021) | Abstract and title |
| 41.(Kefale, Degu et al. 2020) | Title, outcome and study population |
| 42.(Mekonnen, Alhawassi et al. 2018) | Study area and design |
| 43.(Shitu, Aung et al. 2020) | Title and study area |
| 44.(Agalu, Ayele et al. 2012) | Title and abstract |
| 45.(Alemayehu, Baye et al. 2022) | Title, abstract and outcome |
| 46.(Ali, Ademe et al. 2024) | Title and abstract |
| 47.(Ayanaw, Worede et al. 2023) | Title and outcome |
| 48.(Mekonnen, McLachlan et al. 2016) | Abstract and title |
| 49.(Fekadu, Dugassa et al. 2020) | Title |
| 50.(Mekonnen, Yenealem et al. 2019) | Title and outcome |
| 51.(Shitemaw, Jemal et al. 2020) | Outcome and title |
| 52.(Yibrah and Damtie 2015) | Title, abstract and outcome |
| 53.(Kassahun, Abate et al. 2022) | Study population, title |
| 54.(Shashamo, Yesera et al. 2023) | Title, outcome and abstract |
| 55.(Wami, Demssie et al. 2016) | Abstract and title |
| 56.(Afework, Tamene et al. 2023) | Title and outcome |
| 57.(Bekelcho, Birgoda et al. 2024) | Title and abstract |
| 58.(Toitole, Danaso et al. 2024) | Study population and abstract |
| 59.(Feleke, Mulatu et al. 2015) | Title and abstract |
| 60.(Nzayinambaho, Murekatete et al. 2024) | Study area |
| 61.(Hareru, Negassa et al. 2022) | Study population and title |
| 62.(Ayanaw, Worede et al. 2023) | Title and abstract |
| 63.(Sahilu, Getachew et al. 2020) | Title and study outcome |
| 64.(Yohannes, Lessa et al. 2019) | Study population and title |
| 65.(Abegaz and Gebremedhin 2019) | Outcome and title |
| 66.(JIMMA 2018) | Title and abstract |
| 67.(Oweidat, Al-Mugheed et al. 2023) | Study area and outcome |
| 68.(Gizaw, Hailu et al. 2018) | Title and abstract |
| 69.(Ersulo, Yizengaw et al. 2022) | Title and outcome |
| 70.(Zeyede, Jemaneh et al. 2024) | Study population, design and title |
| 71.(Gore 2020) | Title and abstract |
| 72.(Garuma, Woldie et al. 2020) | Title |
| 73.(Fute, Mengesha et al. 2015) | Title and outcome |
| 74.(Tolera, Weldesenbet et al. 2024) | Title |
| 75.(Kasaye, Beshir et al. 2022) | Title and abstract |
| 76.(Awol, Birhanu et al. 2020) | Title and outcome |
| 77.(Abore, Debiso et al. 2022) | Title and abstract |
| 78.(Walle, Shibabaw et al. 2023) | Study design and title |
| 79.(Mekonnen, Yenealem et al. 2019) | Title, study population and abstract |
| 80.(Obse and Ataguba 2020) | Title |
| 81.(Bezu, Seifu et al. 2014) | Title and abstract |
| 82.(Angamo, Chalmers et al. 2018) | Study population and title |
| 83.(Sendekie, Netere et al. 2023) | Study population, outcome and title |
| 84.(Ermias, Gurmesa et al. 2011) | Study design and population and title |
| 85.(Tsega, Habtamu et al. 2024) | Title, design and population |
| 86.(Yadesa, Kitutu et al. 2021) | Study area, title and outcome |
| 87.(Haile, Ayen et al. 2013) | Title, area and abstract |
| 88.(Ersulo, Yizengaw et al. 2022) | Title and abstract |
| 89.(Birhane, Islam et al. 2021) | Design, title and population |
| 90.(Eshetie, Hailemeskel et al. 2015) | Outcome of the study and title |
| 91.(Ejeta, Aferu et al. 2021) | Study population and title |
| 92.(Ayele and Tesfaye 2021) | Title and study outcome |
| 93.(Birarra, Heye et al. 2017) | Study population, abstract and title |
| 94.(Belachew, Erku et al. 2016) | Title |
| 95.(Merid, Gezie et al. 2019) | Title and abstract |
| 96.(Garedow, Mulisa Bobasa et al. 2019) | Title and outcome |
| 97.(Gebremeskel, Gebreyowhans et al. 2021) | Study population and title |
| 98.(Shararo, Asebe et al. 2022) | Title and population of study |
| 99.(Bogale, Tenaw et al. 2022) | Title |
| 100.(Tadele, Hiruy et al. 2016) | Design, population and title |
| 101.(Kemal, Shewaga et al. 2022) | Title |
| 102.(Shegena, Nigussie et al. 2022) | Title and outcome |
| 103.(Ejigu, Gehzu et al. 2018) | Title and abstract |
| 104.(Sisay and Wami 2021) | Title and population |
| 105.(Dagnaw, Indracanti et al. 2024) | Title |
| 106.(Gebremariam, Sema et al. 2024) | Title and abstract |
| 107.(Kushemererwa, Nuwagira et al. 2023) | Study area and title |
| 108.(Anbessa, Hawulte et al. 2024) | Title |
| 109.(Bahta, Berhe et al. 2020) | Study area and title |
| 110.(Engeda 2016) | Included in this systematic review |
| 111.(Eshete, Tesfaye et al. 2021) | Included in this systematic review |
| 112.(Agegnehu, Alemu et al. 2019) | Included in this systematic review |
| 113.(Yalew and Yitayew 2021) | Included in this systematic review |
| 114.(Kefale, Tefera et al. 2017) | Included in this systematic review |
| 115.(Gidey, Seifu et al. 2020) | Included in this systematic review |
| 116.(Kassa Alemu and Biru 2019) | Included in this systematic review |
| 117.(Gurmesa and Dedefo 2016) | Included in this systematic review |
| 118.(Shanko and Abdela 2018) | Included in this systematic review |
| 119.(Hailua, Bhagavathulab et al. 2014) | Included in this systematic review |
| 120.(Zimamu, Agimas et al. 2021) | Included in this systematic review |
| 121.(Kassa, Mulu et al. 2017) | Included in this systematic review |
| 122.(Nadew, Beyene et al. 2020) | Included in this systematic review |
| 123.(Bule, Hamido et al. 2016) | Included in this systematic review |
| 124.(Asefa, Dagne et al. 2021) | Included in this systematic review |
| 125.(Bifftu, Dachew et al. 2016) | Included in this systematic review |
| 126.(Jember, Hailu et al. 2018) | Included in this systematic review |
| 127.(Jifar and Ketebo 2022) | Included in this systematic review |
| 128.(Siraj, Shafi et al. 2022) | Included in this systematic review |
| 129.(Necho and Worku 2014) | Included in this systematic review |
| 130.(Seid, Kasahun et al. 2018) | Included in this systematic review |
| 131.(Abay and Dires 2008) | Included in this systematic review |
| 132.(Shemsu, Dechasa et al. 2024) | Included in this systematic review |
| 133.(Tariku, Mulisa et al. 2015) | Included in this systematic review |
