# Supplementary material for: Prevalence and its associated factors of medical error reporting among healthcare professionals in Ethiopia: Systematic review and meta-analysis
Source: PLoS One. 2025 Jun 2;20(6):e0325114. doi: 10.1371/journal.pone.0325114 (PMC12129177; doi:10.1371/journal.pone.0325114)
Supplement: S4 File — (DOCX) [file pone.0325114.s004.docx]

**Supplementary file 4** revealed the extracted adjusted odds ratio and its lower and upper limits for associated factors in the included articles and data extractors’ name as well as the date of extraction.

| Authors | Prevalence | Predictors | AOR | Lower limits | Upper limits | Data extractor | Date of extraction |
| --- | --- | --- | --- | --- | --- | --- | --- |
| Engeda et al(2016) | 25.4 | Training | 2.96 | 1.34 | 6.26 | Yeshiambaw Eshetie | 4/15/24 |
|  |  | Fear of sanction | 0.27 | 0.12 | 0.58 |  |  |
| Agegnehu et al(2017) | 30.4 | Lack of feedback | 0.69 | 0.61 | 0.76 | Melese Kebede | 4/27/24 |
| Eshete et al(2021) | 31.9 | Fear of sanction | 0.45 | 0.22 | 0.90 | Yeshiambaw Eshetie | 4/18/24 |
|  |  | Lack of feedback | 0.29 | 0.13 | 0.66 |  |  |
| Yalew et al(2021) | 12.5 | Training | 3.6 | 1.15 | 11.45 | Yeshiambaw Eshetie | 4/21/24 |
|  |  | Lack of feedback | 0.3 | 1.1 | 0.9 |  |  |
| Kefale et al(2017) | 90.2 |  |  |  |  | Melese Kebede | 4/15/24 |
| Gidey et al(2020) | 32.1 | Training | 7.31 | 3.42 | 15.62 | Melese Kebede | 4/21/24 |
|  |  | Work experience | 0.36 | 0.13 | 0.97 |  |  |
| Kassa et al(2019) | 50 |  |  |  |  | Yeshiambaw Eshetie | 4/25/24 |
| Gurmesa et al(2016) | 38.8 |  |  |  |  | Melese Kebede | 4/17/24 |
| Shanko et al(2018) | 60.6 |  |  |  |  | Melese Kebede | 4/19/24 |
| Hailu et al(2014) | 28.6 |  |  |  |  | Yeshiambaw Eshetie | 4/23/24 |
| Zimamu et al(2021) | 74.8 | Department type | 0.40 | 0.17 | 0.95 | Yeshiambaw Eshetie | 4/27/24 |
| Kassa et al(2016) | 83.3 |  |  |  |  | Melese Kebede | 4/25/24 |
| Nadew et al(2020) | 27.4 | Work experience | 4.59 | 1.21 | 17.4 | Y.E. | 5/01/24 |
|  |  | Gender(sex) | 3.51 | 1.76 | 7.06 |  |  |
|  |  | Educational status | 5.01 | 2.23 | 11.28 |  |  |
| Bule et al(2016) | 29.2 |  |  |  |  | Y.E. | 5/04/24 |
| Asefa et al(2021) | 37.9 | Work experience | 3.93 | 1.11 | 13.85 | Melese Kebede | 5/3/24 |
|  |  | Gender(sex) | 2.91 | 1.45 | 5.85 |  |  |
|  |  | Educational status | 3.27 | 1.61 | 6.66 |  |  |
| Jember et al(2018) | 57.4 | Gender (sex) | 0.27 | 0.17 | 0.45 | Melese Kebede | 5/11/24 |
| Bifftu et al(2016) | 29.1 | Fear of sanction | 0.35 | 0.17 | 0.71 | Yeshiambaw Eshetie | 5/12/24 |
|  |  | Educational status | 1.38 | 1.01 | 11.13 |  |  |
| Jifar et al(2022) | 46.7 | Training | 3.68 | 1.14 | 11.68 | Melese Kebede | 5/13/24 |
| Siraj et al(2022) | 66.2 | Work experience | 2.88 | 1.65 | 5.47 | Melese Kebede | 5/16/24 |
|  |  | Department type | 3.05 | 1.49 | 18.73 |  |  |
| Necho et al(2014) | 16.2 | Training | 1.82 | 1.1 | 3.1 | Yeshiambaw Eshetie | 5/19/24 |
| Seid MA et al(2018) | 49.1 | Training | 3.36 | 1.07 | 10.05 | Melese Kebede | 5/20/24 |
| Abay et al(2008) | 48.2 |  |  |  |  | Yeshiambaw Eshetie | 5/25/24 |
| Shemsu et al (2024) | 28.7 | Training | 2.87 | 1.46 | 6.28 | Yeshiambaw Eshetie | 6/01/24 |
|  |  | Fear of sanction | 0.49 | 0.25 | 0.97 |  |  |
|  |  | Lack of feedback | 2 | 1.05 | 4.01 |  |  |
|  |  | Department type | 5.48 | 1.67 | 17.8 |  |  |
| Mulisa et al (2015) | 30.8 |  |  |  |  | Melese Kebede | 6/3/24 |
