# Supplementary material for: Prevalence and its associated factors of medical error reporting among healthcare professionals in Ethiopia: Systematic review and meta-analysis
Source: PLoS One. 2025 Jun 2;20(6):e0325114. doi: 10.1371/journal.pone.0325114 (PMC12129177; doi:10.1371/journal.pone.0325114)
Supplement: Attachment — Submitted filename: Magnitude.pdf [file pone.0325114.s005.pdf]

This systematic review and meta-analysis addresses an important topic, namely the prevalence and associated factors of medical error reporting among health professionals in Ethiopia. While the study has several strengths, there are major and minor areas that need improvement.

Appropriateness of Study Design

The rationale for including studies on different types of medical error reporting (medication errors, adverse drug reactions, incidents) could be more robust. The authors should elaborate on the similarities and differences between these types and justify their combined analysis.

Clarity and Relevance of Research Question

The introduction mentions different types of medical errors such as "medication errors, adverse reactions, and incidents" but doesn't clarify whether the review will focus on all of them or a specific type. The primary research question, while generally clear, could be more concisely stated. Defining the scope of "medical error reporting" more precisely in the introduction would enhance clarity.

Literature Search and Selection

While the search strategy is comprehensive, the exclusion criteria are not well-justified. Excluding studies due to "confusing results" is subjective and could introduce bias. Specific and objective criteria for excluding studies based on their results should be developed.

> "...Citations without full text, abstracts, reports, systematic reviews, qualitative and randomised trials as well as confusing results of studies were excluded." (page 5)

The statement above is subjective because it does not explicitly state what constitutes "confusing results." This subjectivity could lead to bias in the study selection process.

Risk of Bias Assessment

The authors stated in the methods section of the article that they used the JBI Critical Appraisal Checklist for cross-sectional studies of Systematic Reviews and Research Syntheses to assess the risk of bias in the primary articles included in the review. They go on to state that the JBI critical appraisal checklist has nine criteria/questions and that they assigned each study as a low, moderate, or high risk of bias based on the results obtained. They also stated that high-quality studies were included to maintain the validity of the review. However, the authors did not justify using the JBI Critical Appraisal Checklist, nor did they provide a reference for the checklist. Additionally, the authors did not provide a detailed explanation of how they assigned each study as a low, moderate, or high risk of bias…page 6

The JBI Critical Appraisal Checklist is not defined. Do you mean the “Joanna Briggs Institute Prevalence Critical Appraisal Checklist for Studies Reporting Prevalence Data

Data Extraction and Analysis:

It is essential to recognize that AORs are adjusted for confounders, meaning they represent the effect of an exposure on an outcome after controlling for the influence of other variables. Combining AORs from different studies with varying adjustments in a meta-analysis can be problematic due to the potential for these adjustments to influence the pooled effect estimate.

The manuscript states that adjusted odds ratios (AORs) were extracted, but it's unclear how these were handled in the meta-analysis. AORs are adjusted for confounders, and combining them from different studies with varying adjustments could be problematic.

"The author, year of publication, study year study design, sample size, response rate, prevalence (practice) of error report, odds ratio, lower and upper border of odds ratio of significantly associated factors and area of study were extracted in each study using the data extraction form." (Page 5)……. "Adjusted odds ratio (AOR) was the most commonly reported measure of association in the eligible primary studies and random-effect model was also used to estimate the pooled odds ratio of this study." (Page 6)

These excerpts indicate that the authors extracted AORs from the included studies and used them in the meta-analysis. However, the manuscript does not provide details on how the authors addressed the potential issue of combining AORs that may have been adjusted for different confounders in each primary study. This lack of clarity raises concerns about the appropriateness of the meta-analysis of AORs.

The authors should explicitly state or clarify how they handled the AORs in the meta-analysis. If no specific adjustments were made, they should consider extracting and analysing crude odds ratios, which have not been adjusted for confounders, to avoid potential issues related to combining AORs with varying adjustments.

Clarity and Completeness of Results

The results section lacks a comprehensive presentation of the included studies' characteristics. A table summarizing key features like sample size, study population, prevalence, and quality score would improve clarity. Additionally, the reporting of the meta-analysis results could be enhanced by including measures of heterogeneity for each pooled analysis.

- **Lack of comprehensive presentation of included studies' characteristics:** The paper does not include a comprehensive table summarizing the characteristics of the included studies. The only table provided (Table 1) is a basic list of authors, study area, design, sample size, prevalence, and quality assessments. Important details like the specific types of medical errors studied could be included in the table if available
- **Need for additional measures of heterogeneity:** While the authors mention the I^2 statistic to indicate high heterogeneity in the pooled analyses, they do not report the actual I^2 values or other measures of heterogeneity for each pooled analysis. This information would help the extent of variability between studies.

"A total of 24 studies were included in this review; 5 of them were about incident reporting, 3 of them about medication error reporting and the remaining 16 of them were about adverse drug reaction reporting. All the included studies reported the prevalence of medical error reporting (table.1)."….page 7

"The I^2 value of 0, 25%, 50 and 75% represent no, low, moderate and high heterogeneity respectively."….page 6

"The galpraith plot (fig. 4) and the percentage of I2 statistics of the forest plot revealed the presence of marked heterogeneity among the included primary studies (I2 = 98.79%, p < 0.01)."…page 10

These quotes highlight that the paper acknowledges the different types of medical error reporting and the presence of heterogeneity but does not provide a comprehensive summary of study characteristics or report specific heterogeneity measures for each analysis.

Interpretation of Finding

The discussion section is repetitive in places, particularly regarding the prevalence findings. The comparison to studies from other countries is helpful but could be more concise (Page 17 -18)

- Briefly state the pooled prevalence and then focus on comparing it to the prevalence found in other similar systematic reviews or meta-analyses, rather than individual studies.
- Consider grouping the comparison countries into higher and lower prevalence categories to identify trends.
- Discuss the potential reasons for the observed differences in prevalence between Ethiopia and other regions, considering factors like healthcare system structure, reporting culture, and types of errors included in the studies.

Clarity and Organization

The manuscript contains grammatical errors and inconsistencies. A thorough proofreading and language edit are needed.

Some examples;

- "These errors rise from time to time" (page 2)
- Missing articles - "A similarly study" (page 2)
- "clinicians have also moral" (page 2)
- "retrived" instead of "retrieved" (page 2), "plooled" instead of "pooled" (page 2, page 4, "analye" instead of "analyze" (page 3), "irelevant" instead of "irrelevant" (page 7)
- Incorrect verb tense: "The pooled AOR... was 3.25" (page 12) - should be "is"
- Missing articles: "In the review, eight studies reported..." (page 12) - should be "In the review, **the** eight studies reported..."
- Run-on sentences: "Nurses and pharmacists report medical error than those midwives, medical doctors and laboratorist..." (page 20)………….. needs to be broken down for clarity.
- Inconsistent use of abbreviations "AOR"
