# Supplementary material for: Prevalence and its associated factors of medical error reporting among healthcare professionals in Ethiopia: Systematic review and meta-analysis
Source: PLoS One. 2025 Jun 2;20(6):e0325114. doi: 10.1371/journal.pone.0325114 (PMC12129177; doi:10.1371/journal.pone.0325114)
Supplement: Attachment — Submitted filename: Response to reviewers.pdf [file pone.0325114.s008.pdf]

Point by point response for editor

Journal Name PLOS ONE

Manuscript number: *D-24-27747*

Title*: Prevalence and its associated factors of medical error reporting among healthcare professionals in Ethiopia: Systematic Review and Meta-Analysis*.

Thank you, editor, for the opportunity to submit the revised version of the manuscript and to correct edition problems and respond questions.

Point by point response for reviewer-2 (answers written in bold)

First of all we would like say thank you for your valuable time, expertise, and constructive feedback. We benefited a lot from your insightful comments.

Reviewer's comments or questions

1. Abstract

Issues:

• Clarity and Structure: The abstract still contains grammatical errors and could be more concise.

• Incomplete Information: The abstract mentions that 24 studies were included but does not specify the types of medical errors or the professions of health workers.

Recommendations:

• Revise for Clarity: Proofread the abstract to correct grammatical errors and improve sentence structure.

• Include Specifics: Provide brief information on the types of medical errors studied and the health professions involved.

• Structure: Ensure the abstract follows a clear structure: Background, Methods, Results, and Conclusion.

Authors' response: **Thank you for your valuable feedback and recommendations. We appreciate the time and effort you dedicated to enhancing the grammar, clarity and readability of this manuscript. We have carefully considered and incorporated all of your recommendations. We have carefully corrected the grammar and language editions as suggested. We have added all of your recommendations in the restructured abstract**. Please see page 2.

Reviewer's comments or questions

2. Introduction

Issues:

• Grammar and Clarity: The introduction contains grammatical errors which affect readability.

• Flow and Coherence: The narrative jumps between ideas without smooth transitions, making it difficult to follow.

• Literature Review: While international studies are cited, there is limited critical analysis of existing literature specific to Ethiopia, and the research gap is unclear.

Recommendations:

• Proofread for Language Clarity: Correct grammatical errors and improve sentence structure for better readability.

• Enhance Flow: Use transitional phrases to connect ideas and ensure a logical progression of thought.

• Expand Literature Review:

0 Critical Analysis: Provide a critical synthesis of existing studies on medical error reporting in Ethiopia and globally.

0 Identify Gaps: Clearly articulate the gaps in the literature that your study aims to fill.

0 Justify the Study: Emphasize the importance of a systematic review and meta-analysis to consolidate fragmented findings and provide national-level insights.

Authors' response: We appreciate your thoughtful comments and guidance.

- ✓ **Regarding grammar and clarity: We have thoroughly corrected grammar and language issues in the revised manuscript. Please see the revised version.**
- ✓ **Flow and coherence: We have incorporated transitional phrases to ensure smooth flow ideas and transitions. Kindly see the revised manuscript.**
- ✓ **Literature review: The authors have critically analyzed literature, identified gaps and provided justification for the need for this study. Kindly refer to page 4, lines 22-29.**

Reviewer's comments or questions

3. Methods

Issues:

• Search Strategy: The description lacks detail about search terms, specific inclusion/exclusion criteria, and the search timeframe.

• Data Extraction and Quality Assessment: The process is briefly described but could benefit from more detail, including how reviewer disagreements were resolved.

• Risk of Bias Assessment: There is limited discussion on how the quality of the included studies was assessed using the JBI checklist.

Recommendations:

• Provide Detailed Search Strategy: Include the specific search terms used, the time frame of the search, and any language restrictions.

• Inclusion/Exclusion Criteria: Specify the criteria used to include or exclude studies, such as

study design, population, outcomes measured, and settings.

• Data Extraction Process: Elaborate on the data extraction process, including how data were managed and stored and how discrepancies were handled.

• Quality Assessment: Provide more detail on how the JBI checklist was applied, including the criteria assessed and how the quality scores influenced the inclusion of studies.

• Subgroup Analysis: In the methods and results, provide more detail on the subgroup analyses conducted and discuss their implications.

• Statistical Methods: Provide more detailed descriptions of the statistical methods used, including how the random-effects model was applied and how heterogeneity was quantified and addressed.

***Authors' response*:** Thank you for your insightful and constructive feedback.

- ✓ **Regarding the search strategy: we have implemented a comprehensive approach by combining MeSH terms and free-text keywords. The search string was constructed using Boolean operators. Kindly refer to page 5, lines 14-23.**
- ✓ **Inclusion and exclusion criteria: We have provided a detailed description of the eligibility criteria, clearly specifying the study design, context, condition and population. Please see page 5, lines 9-13.**
- ✓ **Data extraction process: The authors have provided a more detailed explanation of the data extraction process, including how data stored and how disagreements were handled, in the revised manuscript. Kindly refer to page 6, lines 2-6**
- ✓ **Quality assessment: In the revised manuscript, we have clarified how the JBI checklist was used to assess the methodological quality of each study, as well as how the quality scores influenced the inclusion of articles in the review. Please see page 6, lines 17-24.**
- ✓ **Subgroup analysis: We performed a subgroup analysis based on the area of study and types of medical error reporting; however, we were unable to precisely identify the source of heterogeneity. This limitation has been acknowledged in the manuscript. Please see page 9, lines 15-26.**

- ✓ **Statistical methods: We have provided more descriptions about the statistical methods. A random-effects model was used to estimate the pooled prevalence of medical error reporting, particularly when high heterogeneity observed in the included studies. We have also clarified how heterogeneity was quantified using I^2 statistic. Both subgroup and sensitivity analyses were performed to identify sources of heterogeneity and assess the robustness of study. Please see page 7, 8 and 9.**

Reviewer's comments or questions

4. Results

Issues:

• Presentation of Findings: The results section could benefit from more explicit organisation and detailed descriptions of the included studies.

• Heterogeneity and Publication Bias: While heterogeneity is acknowledged, the implications are not fully discussed. Methods used to address heterogeneity (e.g., subgroup analysis) need more explanation.

• Associated Factors: The analysis of associated factors is presented but lacks depth in interpretation.

Recommendations:

• Organise Results with Subheadings: Use subheadings such as 'Study Selection,' 'Characteristics of Included Studies,' 'Pooled Prevalence,' 'Heterogeneity Assessment,' 'Publication Bias,' 'Sensitivity Analysis,' 'Subgroup Analysis,' and 'Factors Associated with Medical Error Reporting.'

• Discuss Heterogeneity: Provide a more detailed discussion on the high heterogeneity observed, potential reasons, and how it was addressed in the analysis.

• Interpret Associated Factors: Offer in-depth interpretation of the findings related to associated factors, including potential mechanisms and implications.

Authors' response**: Thank you for your suggestions. We greatly appreciate your feedbacks, which has helped improve the clarity of this study.**

- ✓ **Regarding presentations of finding: we have incorporated your suggestions in the revised manuscript. Please see page 7, 8, 9, and 10.**

- ✓ **Heterogeneity and publication bias: the authors acknowledge the variability among the included studies and conducted a subgroup analysis (type of medical error reporting and area of study) to investigate the source of the heterogeneity. While the subgroup analysis based on the study area identified the source but it did not fully determine the cause of heterogeneity. We acknowledged this in the limitation and recommended for future research. Please see page 9 and 13.**
- ✓ **Associated factors: we have incorporated your suggestions and made the necessary corrections based on the recommendations. Please see page 9 and 10.**

Reviewer's comments or questions

5. Discussion

Issues:

• Interpretation of Results: The discussion provides comparisons with other countries but lacks depth in exploring the reasons behind the differences.

• Link to Existing Literature: The study findings are limitedly integrated with existing literature, particularly in explaining why certain factors are associated with medical error reporting.

• Limitations: The limitations section is brief and does not fully consider the impact of the study design and heterogeneity.

Recommendations:

• Deepen Interpretation: Explore potential reasons for Ethiopia's low prevalence of error reporting, considering cultural attitudes, systemic barriers, and policy implications.

• Integrate with Literature: Discuss how your findings align or contrast with previous studies within Ethiopia and internationally and what this means for understanding medical error reporting.

• Expand on Limitations: Provide a thorough discussion of the study's limitations, including the high heterogeneity, potential publication bias, reliance on self-reported data, and the limitations inherent in cross-sectional studies.

Authors' response: **Thank you for your constructive suggestions and comments.**

- ✓ **Regarding interpretations of results: we have expanded the discussions in the revised manuscript to further explore the reasons behind the low reporting in the country. Please see page 11 and lines 1-17.**

- ✓ **Integrate with literature: we have discussed our findings in relation to previous systematic review and meta-analysis conducted globally. Since systematic reviews and meta-analyses provide stronger evidence than primary studies, we focused on comparing and contrasting our results with these reviews, rather than primary articles. However, due to lack of systematic reviews we discussed the results with primary studies in some extent. Please see page 11 and 12.**
- ✓ **Limitations: Based on your comments and suggestions, we have broadened the limitations section. Please refer to page 13, lines 12-19**.

Reviewer's comments or questions

6. Conclusion

Issues:

• Clarity and Impact: The conclusion restates the findings but could be more impactful by emphasising practical implications and specific recommendations.

Recommendations:

• Emphasise Practical Implications: Highlight how the findings can inform policy and practice, suggesting specific interventions tailored to the Ethiopian context.

• Call to Action: Encourage stakeholders to implement strategies to improve medical error reporting, such as policy changes, training programs, and developing non-punitive reporting cultures.

***Authors' response*: Thank you for your constructive feedback and suggestions. We have incorporated your suggestions into the revised manuscript and have rewritten the conclusion as advised.** Please refer page 13, lines 21-29.

Reviewer's comments or questions

7. Language and Grammar

Issue:

• The manuscript contains grammatical errors, typos, and awkward phrases that affect readability.

***Authors' response*: Thank you once again for your valuable suggestions and thorough review. We have made efforts to correct the grammatical errors, typos and inappropriate phrases in the manuscript. Kindly review the revised version.**

Reviewer’s comments or questions

8. Formatting and References

Issues:

• Reference Style: The references are inconsistently formatted and may not adhere to the Vancouver style applied throughout the manuscript.

• Citations: Some in-text citations lack corresponding references, and vice versa. ***Authors’ response*****: We apologize for not using the Vancouver referencing style consistently throughout the reference list. We used Google scholar to cite the articles and have made efforts to correct the font and punctuation in the revised manuscript. Additionally, we added the missing citations in the text and removed references from reference lists that were not cited in the text. Please see the reference section in the revised manuscript.**

Point by point response for reviewer-3(answers written in bold)

First of all we would like say thank you for your valuable time, expertise, and constructive feedback. We benefited a lot from your insightful comments.

Reviewer's comments or questions

① Language Editing:

The manuscript requires extensive language editing to improve clarity, grammar, and readability.

***Authors' response*: We thank you for your extensive review and valuable feedback. Your concern regarding to the language and clarity of the manuscript is critical. We have thoroughly revised the manuscript to address these issues related to grammar, clarity and readability. Please check the revised manuscript.**

② Abstract: The abstract should be more concise:

1. The introduction should provide the background in 1-2 sentences and clearly state the study's objective. Avoid repeating the objective in both the introduction and methods sections. The authors should focus on using the word limit of the abstract effectively.
2. In the Methods section, specify the duration of the database search and the types of studies included.
3. In the Results section, mention the total number of records identified through the database and manual search.
4. The results are not precise. For example,

- Clarify what is meant by "training"—does it refer to a lack of training on medical error reporting?
- Specify the context of "work experience" and "education" to avoid ambiguity.
- Clearly state which sex is being referred.

***Authors' response*: Thank you for insightful comments. We have made it more concise as recommended and placed the objectives in the methods section.**

We have also specified the database search duration and types of studies in the revised abstract. The total numbers of records identified through databases have also mentioned.

We intended to say that professionals who got training about medical error reporting. The authors have specified as having increased work experience and higher educational status in the revised manuscript. Kindly refer to page 2.

Finally, we meant being female healthcare professionals significantly associated with increased medical error reporting.

Reviewer's comments or questions

③ Introduction:

1. The introduction needs to be more concise and well-structured. Ensure the flow of information is logical, and avoid repeating the same content across paragraphs (notably in paragraphs 1-3).

2. In Paragraph 1, provide numeric evidence (preferably global data) to substantiate the statement: "Even though the majority of errors do not lead to an apparent adverse effect, a considerable number of patients either suffer permanent harm or die every year worldwide as a result of these mistakes."

3. The statement (paragraph 4)"Despite the numerous advantages of error reporting, the magnitude of underreporting remains high in different countries around the world" is supported with data from only two countries. Including global-level data or examples from diverse regions would enhance the credibility and comprehensiveness of this claim.

***Authors' response*: Thank you for your constructive comments. The revised manuscript has made more concise and well-structured. We apologize for the redundancy of ideas and incoherency. We acknowledged concerns and we made corrections in the revised version. Please see page 3 and 4.**

The authors have also added numeric evidence on the statement in paragraph 1. Please see page 3 and line 8-9.

We have added evidence from different countries on the statement in paragraph 4. Please see page 4 and lines 5-10.

Reviewer's comments or questions

④ Methods:

1. Provide details of the search strategy used for at least one database to improve transparency and replicability.

2. To enhance clarity and a structured overview of the inclusion and exclusion criteria, include a table summarizing the PICOS criteria (Population, Intervention, Control, Outcome, and Study

design).

3. The study aims to determine the pooled prevalence of medical error reporting and identify associated factors among health professionals in Ethiopia. The authors should justify why studies published in the national languages of Ethiopia were not included

Authors' response: **Thank you for your insightful suggestions. We have included the search string in the revised manuscript and attached a supplementary file about the CoCoPoP design for clarity. Please see page 5 and lines 15-24.**

We acknowledge your comment regarding medical error report studies conducted and published in the national languages of Ethiopia. We conducted a search of articles from databases without language restriction; however, we did not find any studies conducted and published in the national languages of Ethiopia among the identified articles. So, we have corrected Ethiopian languages restrictions in the revised manuscript. Please see page 5, line 7.

Reviewer's comments or questions

⑤Results:

1. The authors use the terms 'sex' and 'gender' interchangeably. These terms should be unified throughout the manuscript to avoid confusion.

2. Even the results section does not clearly explain how sex is associated with medical error reporting (was the prevalence higher among men or women?)

3. The term "education level" is unclear. If the authors are referring to the professional category (e.g., nurses, physicians, etc.), it would be more appropriate to use this terminology rather than dividing participants into educational categories.

Authors' response: **Thank you for constructive feedback.**

1. **As suggested, we have used the term 'gender' consistently throughout the revised manuscript.**
2. **In this study, we found that being a female healthcare professional was significantly associated with medical error reporting, indicated that women are more likely to report error than men. Please see page 10 and lines 20-23.**

3. **Both educational status and professional category were statistically associated with medical error reporting in the primary studies included in this review. However, when the data were pooled, professional category (type of disciplines) was not significantly associated with medical error reporting. Therefore, we did not include the pooled AOR for professional categories in the result section. Instead we discussed it in the discussion section, as this factor was significantly associated with medical error reporting in three of the included studies. Finally, we meant educational status not professional categories (type of disciplines).**

⑥Discussion:

1. Include a section at the end of the discussion offering recommendations for future research and practical implications. Authors could add a discussion on the current legislation and policies related to medical error reporting in Ethiopia, highlight any loopholes or gaps in the system, and propose methods to improve error reporting practices.

***Authors' response*: Thank you for your thoughtful suggestions. As recommended, we have added a dedicated section for recommendations for future research and practical implications. Please see page 14, lines 2-13. Additionally, we have incorporated a discussion on the current legislation and policy related to medical error reporting in Ethiopia; highlighting the existing gaps in the system. We have also proposed potential methods to improve error reporting practices in the country.** Please see page 12 and lines 3-7.
