# Supplementary material for: Prevalence and its associated factors of medical error reporting among healthcare professionals in Ethiopia: Systematic review and meta-analysis
Source: PLoS One. 2025 Jun 2;20(6):e0325114. doi: 10.1371/journal.pone.0325114 (PMC12129177; doi:10.1371/journal.pone.0325114)
Supplement: Attachment — Submitted filename: Response to reviewers.docx [file pone.0325114.s007.docx]

***Point by point response for editor(written in bold)***

***Journal Name PLOS ONE***

***Manuscript number:*** *D-24-27747*

***Title****: Prevalence and associated factors of medical error reporting among healthcare professionals in Ethiopia. Systematic review and meta-analysis*.

**Thank you, editor, for the opportunity to submit the revised version of the manuscript and to correct edition problems of language as well as grammar**. We would like to give response to question which is not raised in the reviewers’ comment by assuming it is editor’s question

Question

The primary research question, while generally clear, could be more concisely” stated. Defining the scope of "medical error reporting" more precisely in the introduction would enhance clarity.

***Authors’ response: Regarding the need of defining the scope of medical error reporting we agree that a clearer explanation improves the reader’s understanding and enhance overall clarity of the review. We have incorporated your valuable comments in our revised manuscript (see page 3, line-16 and 17).***

***Point by point response for reviewer-1(answers written in bold)***

First of all we would like say thank you for your valuable time, expertise, and constructive feedback. We benefited a lot from your insightful comments.

**Reviewer’s comments or questions**

“The rationale for including studies on different types of medical error reporting (medication errors, adverse drug reactions, incidents) could be more robust. The authors should elaborate on the similarities and differences between these types and justify their combined analysis.”

***Authors’ response****:* ***We thank you for your insightful input and concern regarding the inclusion of various types of medical error reporting and the rationale of the combined analysis. We acknowledge your comment on the need for robust explanation on the similarities and differences among medication error reporting, adverse drug reaction reporting and incident reporting (see page a3 and 4****).* ***Even though, these medical error reporting categories looks like have slight distinction, they are almost similar in their goals of reporting, advantages, causes of occurrence, and responsibility of disclosing them****.* ***We incorporated in the revised manuscript (see page 3 and 4****).* ***To name few:-***

**1. *Diversity of error reports*:** All adverse drug reaction reporting, medication error reporting and incident reporting allows healthcare managers to capture a comprehensive view of patient safety issues at the hospital level. Each type can reveals different dimensions of healthcare challenges in each working unit and department of healthcare providers.

**2. *Interconnectedness*:** These types of errors are often interconnected. For instance, a medication error may lead to an adverse drug reaction and both can be categorized as an incident affecting patient safety culture that leads reduction in patient outcome of the healthcare institutions. Therefore, analyzing them together can identify patterns and systemic vulnerabilities of the healthcare delivering institution rather than focusing each fragmented types of error reporting.

**3*. Regarding report responsibility:*** Doctors, nurses, and midwives are often reporting medication error and adverse drug reactions as part of their clinical responsibilities and pharmacists also play a crucial role in identifying and reporting medication errors as well as adverse drug reactions. Thus, there is role overlapping rather than distinction among healthcare professionals that reveals the need of using a comprehensive error reporting system.

**4. *Justification of the combined analysis***: This combined analysis emphasize the benefit of holistic approach to common root cause and developing effective strategies for prevention that can be applied across these different type of errors, and used to bring quality cares in the organization as whole. This combined analysis employed for regulation and accreditation standard preparations by considering healthcare institution as one entity rather than various departments separately.

**5*. Implication***: Understanding these errors collectively can reveal need of police change in this sector aimed at improving healthcare services provided and reducing unwanted events.

***Revision in the manuscript*:** We incorporated these clarifications and justifications in the revised manuscript to offer more robust rationale for the combined analysis.

**Reviewer’s comments or questions**

The authors need to rephrase the research question to be more focused for example "What is the prevalence of medical error reporting among health professionals in Ethiopia, and what are the associated factors?”

***Authors’ response****:*	***We modified the research questions and title as directed by reviewers.***

**Reviewer’s comments or questions**

The author should consider providing specific, objective criteria for excluding studies based on their results.

***Authors’ response****:* ***Thank you again for your constructive comments regarding the exclusion criteria. We appreciated your concern about the phrase “confusing results” lacks clarity and could introduce bias and subjectivity. We revised the criteria to offer specific and objective parameters to enhance clarity and reduce potential bias in the study selection process (see page 6, line 1 and 2).***

**Reviewer’s comments or questions**

What is the author's rationale for using the JBI Critical Appraisal Checklist for prevalence studies?

***Authors’ response****:* ***Thank you for your thorough review and constructive inputs on the use of JBI critical appraisal Checklist. We apologize for the oversight in not providing a justification and reference for this checklist. We clarified that we were indeed referring to the “Joanna Briggs Institute Prevalence Critical Appraisal Checklist for Studies Reporting Prevalence Data.” We included the appropriate citation in the revised manuscript. Moreover, we provided a detailed explanation of how we assigned each article as low, moderate, or high risk of bias based on this checklist (see page 7 and 8).***

**Reviewer’s comments or questions**

There is a need to clarify the approach to analyzing AORs in the meta-analysis, considering potential issues related to combining adjusted effect estimates from different studies.

***Authors’ response: Thank you again for your comment regarding the unclearness of handling the adjusted odds ratios (AOR) in this meta-analysis. We acknowledged your concern about the potential issues rising from combining AORs with varying adjustments for confounders across different studies. We incorporated strategies in the revised manuscript to handle AORs with varying adjustments of confounders among the included studies. To list them:-***

***Random effect model: Employing random effect model can effectively account for the variability in adjusted odds ratio (AOR) among the studies included in the meta-analysis. The model gives consideration for weighted average, the true effect size vary among the included articles rather than assuming a single true effect size, accounting for between-study variability, flexibility in adjustment, exploration of heterogeneity etc. Therefore, using this model helps in handling varying adjustment of AORs in meta-analysis by accounting for the inherent heterogeneity among the included studies, leading to a more reliable and comprehensive of the overall effect.***

***Sensitivity analysis: we conducted sensitivity analysis by excluding articles with specifically different adjustments (work experience, educational status) and using alternative models to see the robustness of the pooled result. Therefore, based on the sensitivity analysis there is no study with particularly different adjustment made disproportionally affecting the pooled results.***

***During data extraction and Quality assessment: we performed robust quality assessment to manage variability in AORs by ensuring that only studies with robust methodologies and appropriate adjustments included*** *[(training, fear of administrative sanction, and lack of feedback all are answered by using Yes or No****), gender (male or female)] most of the included articles had similar adjustment except work experience. Thus, this revealed robustness and reliability of the pooled results of the study*.**

**Reviewer’s comments or questions**

There is no table summarizing the characteristics of included studies and reported heterogeneity measures for each pooled analysis.

***Authors’ response****:*

***We would like say thank you for your valuable inputs. We agree that the results section would benefit from a more comprehensive presentation of the included studies' characteristics. Incorporating this comprehensive summary in a meta-analysis enhances transparency, allows for easier comparison of studies, and helps readers understand the context and relevance of the findings. We apologize for some missed value of actual I² and other heterogeneity measures for each pooled value in the subgroup analysis. These are incorporated in the revised manuscript (see page 9, line 9-19).***

***Point by point response for reviewer-2(answers written in bold)***

First of all we would like say thank you for your valuable time, expertise, and constructive feedback. We benefited a lot from your insightful comments.

**Reviewer’s comments or questions**

1. Clarity and Language:

The manuscript suffers from frequent grammatical errors, awkward phrasing, and incorrect word usage (e.g., “professional sex” instead of "gender"). These issues detract from the readability and professionalism of the paper.

Some sentences are confusing, and the logic is difficult to follow in parts, particularly in the introduction and discussion sections. For example, the line “Medical error is a failure of a ‘planned action to be completed as intended or the use of a wrong plan to achieve aim’” is not clearly articulated.

Recommendation: The manuscript should undergo thorough language editing for clarity, grammar, and word choice

***Authors’ response: Thank you for your constructive comment. We appreciate your thorough feedback on the grammatical errors and inconsistencies in the manuscript. Your careful review underscores the necessity for thorough proofreading and language editing, which we addressed in the revised manuscript (see the revised manuscript).***

2. Methodological Details:

**Data Extraction**: While the authors mention using a data extraction form, they provide limited details about how reviewer disagreements were handled. A more thorough explanation of the steps taken to resolve conflicts would enhance the robustness of the review process.

***Authors’ response: We incorporated the explanations of handling disagreements occurring during data extraction (see page-7, line 1-5).***

**Risk of Bias**: The authors mention using the JBI Critical Appraisal Checklist but do not detail how the included studies were assessed. For example, it is unclear if the analysis weighted the low-risk, moderate-risk, and high-risk studies differently.

Recommendation: Include a table with the quality assessments of all included studies and explain how potential biases were accounted for in the meta-analysis.

***Authors’ response****:* ***Thank you for your thorough review and constructive inputs on the use of JBI critical appraisal Checklist. We apologize for the oversight in not providing a justification and reference for this checklist. We clarified that we were indeed referring to the “Joanna Briggs Institute Prevalence Critical Appraisal Checklist for Studies Reporting Prevalence Data.” We included the appropriate citation in the revised manuscript. Moreover, we provided a detailed explanation of how we assigned each article as low, moderate, or high risk of bias based on this checklist (see page 7 and 8).***

**3. Discussion and Interpretation**:

The discussion would benefit from more profound reflection on the implications of the findings, particularly in the global context. Comparisons are made to Kenya, Nigeria, and other countries, but the reasons for the differences are not sufficiently explored. Additionally, the implications of the findings for health policy in Ethiopia are not discussed in depth.

Causality: While several factors (e.g., training, fear of administrative sanctions) are identified as being associated with reporting behavior, the authors should clarify that causality cannot be inferred from the cross-sectional studies included in the meta-analysis.

Recommendation: Expand the discussion to address the implications of the findings more comprehensively, considering health policy and the practical applications of the results.

***Authors’ response: Thank you for your thorough observation on this section and we realized some parts were repetitive particularly in the title. Thus, based on your comment we avoided the redundant and corrected the title of the study more succinctly in the revised manuscript. Additionally, we appreciated your suggestion regarding comparison of this finding with similar systematic review findings and group countries into higher or lower prevalence categories. Even thus, the result of this study ought to compare with the results of systematic review studies, we are forced to compare it with the results of primary studies due to lack of literatures on this topic. We have also included a discussion of the potential reasons contributing to the differences in prevalence between Ethiopia and other regions in the revised manuscript, considering elements such as healthcare system structure, reporting culture, and the types of errors addressed in the studies (see page 12-14).***

4**. Heterogeneity and Subgroup Analyses:**

The heterogeneity (I2 = 98.79%) is very high, indicating significant variability among the studies. Although the authors conduct subgroup analyses, the explanation of the sources of heterogeneity could be more detailed. For instance, the potential impact of different healthcare settings, reporting systems, or healthcare worker roles on heterogeneity is not fully explored.

Recommendation: Provide a more in-depth discussion of the possible causes of heterogeneity and whether future research should target these areas to reduce variability.

***Authors’ response: Thank you for your thorough view. We addressed your points in the revised manuscript (see page 10 and 11).***

5. Figures and Tables:

While the forest plots and subgroup analyses are helpful, the presentation of the data could be more organized. For instance, Table 1, which presents the characteristics of included studies, is somewhat cluttered and challenging to read.

Recommendation: Consider reformatting the tables to improve clarity and readability

***Authors’ response****:* ***We would like say thank you for your valuable inputs. We agree that the results section would benefit from a more comprehensive presentation of the included studies' characteristics. Incorporating this comprehensive summary in a meta-analysis enhances transparency, allows for easier comparison of studies, and helps readers understand the context and relevance of the findings. We apologize for some missed value of actual I² and other heterogeneity measures for each pooled value in the subgroup analysis. These are incorporated in the revised manuscript (see page 9, line 9-19).***
